# Comprehensive transcriptome and proteome analysis revealed the molecular mechanisms of melatonin priming and waterlogging response in peach

**DOI:** 10.3389/fpls.2025.1527382

**Published:** 2025-02-07

**Authors:** Xianbin Gu, Linghong Lu, Jing Gao, Fei Fan, Genhua Song, Huiqin Zhang

**Affiliations:** Institute of Horticulture, Zhejiang Academy of Agricultural Sciences, Hangzhou, China

**Keywords:** *Prunus persica*, waterlogging, melatonin, transcriptome, proteome

## Abstract

Waterlogging substantially hampers the growth and development of plants. The escalating trajectory of global climate change is heightening both the frequency and intensity of waterlogging events. Peach trees are particularly vulnerable to waterlogging, with the resultant hypoxia in the rhizosphere profoundly influencing their growth and productivity. This study explored the responses of peach seedlings to waterlogging and the regulatory effects of melatonin priming. After a 24-h waterlogging treatment, a significant increase in relative electrical conductivity and an accumulation of reactive oxygen species were observed, ion permeability was markedly alleviated by melatonin priming. Transcriptomic and proteomic analyses were conducted on peach root samples to elucidate the molecular mechanisms involved in the response to waterlogging and melatonin priming. Transcriptome analysis implicated genes related to ‘DNA-binding transcription factor activity’, such as AP2/ERF, HSF and WRKY transcription factors, in response to waterlogging. The glycolysis/gluconeogenesis pathway was also significantly enriched, indicating its critical role in the metabolic response to waterlogging. A correlation analysis between differentially expressed genes and proteins highlighted the regulation of numerous genes at both the transcriptional and translational levels. Furthermore, core DEGs/DEPs, including heat shock proteins and stress-related proteins, were identified. Notably, ERF VII member ERF071 (Prupe.8G264900), ADH (Prupe.8G018100), and PCO (Prupe.7G011000) emerged as potential targets for genetic manipulation to enhance waterlogging tolerance in peach. This research provides targets for breeding waterlogging-tolerant varieties and strategies to mitigate waterlogging stress in peach.

## 1 Introduction

Waterlogging constitutes a significant abiotic stress that profoundly impacts plant growth, development, and agricultural productivity. This stress typically results from excessive rainfall, flooding, or inadequate soil drainage, leading to oxygen deficiency in the soil and rhizosphere (Kreuzwieser and Rennenberg, 2014). Amidst global climate change, the frequency and severity of extreme weather events, such as powerful typhoons and torrential rains, are on the rise, leading to more frequent and severe waterlogging episodes (Zhou W. et al., 2020). This has emerged as a serious threat to various plant species. The influence of global climate change on waterlogging is considerable. Rising temperatures can modify precipitation patterns, resulting in more frequent and intense rainfall in some regions, while others may endure prolonged droughts followed by heavy rainfall (Tabari, 2020). These fluctuations, in conjunction with changes in soil moisture and temperature, can exacerbate waterlogging conditions. For instance, in agricultural regions prone to rhizosphere hypoxia, the risk of soil waterlogging is anticipated to rise due to these climatic alterations (Tian et al., 2021). Waterlogging can elicit a spectrum of deleterious effects on plants, including reduced photosynthesis, decreased stomatal conductance, lower leaf chlorophyll content, hormonal imbalances, and compromised water and mineral uptake (Loreti et al., 2016). These impacts highlight theocratical need for strategies to mitigate the effects of waterlogging on plant health and agricultural systems. Fruit trees, such as apple, peach, and citrus, are particularly vulnerable to waterlogging. The tolerance of fruit trees to hypoxic conditions varies by species and cultivar. It is also influenced by factors such as plant age, developmental stage, soil type, and season (Habibi et al., 2023). Waterlogging has become one of the primary abiotic stresses limiting fruit productivity globally.

Phytohormones play a critical role in the plant response to rhizosphere hypoxia and serve as key regulators to overcoming stress conditions. Melatonin, a naturally occurring compound, has attracted attention as a potential modulator of plant stress responses, including those to waterlogging (Jahan et al., 2021). This molecule is implicated in numerous physiological processes in plants, such as scavenging reactive oxygen species (ROS), augmenting antioxidant enzyme activity, and stimulating photosynthesis (Sati et al., 2023; Wang X. et al., 2024). Recent studies suggested that the application of exogenous melatonin can enhance plant tolerance to waterlogging by regulating their antioxidant metabolism, nitrogen uptake, and the composition of the rhizosphere microbial community. For instance, in apples, melatonin facilitates post-waterlogging recovery by modulating the structure and function of the rhizosphere microbiome, bolstering antioxidant capacity, and optimizing nitrogen absorption and utilization (Cao et al., 2024a; Cao et al., 2024b). The mitigating effects of melatonin pretreatment on the adverse impacts of waterlogging stress have also been observed in other species, such as wheat (Ma et al., 2022) and peach (Gu et al., 2021).

Transcriptome and proteome analyses have yielded invaluable insights into the molecular mechanisms by which plants respond to waterlogging stress. These investigations have identified a multitude of differentially expressed genes (DEGs) and associated pathways that are integral to key processes such as respiration, carbohydrate metabolism, photosynthesis, phytohormone metabolism, ROS metabolism, mineral uptake, and protein and amino acid metabolism under hypoxic conditions (Lin Y. et al., 2019; Zeng et al., 2019; Li et al., 2022). A thorough comprehension of the physiological, biochemical, and molecular responses of plants to waterlogging is essential for formulating strategies to enhance their tolerance to this abiotic stress. By delineating these responses and identifying potential targets for genetic improvement, significant advancements can be made in the development of more resilient plant varieties and sustainable agricultural practices. This is particularly pertinent in light of rising frequency of waterlogging events associated with climate change, underscoring the imperative for innovative strategies to safeguard agricultural sustainability and food security.

Peach trees are highly susceptible to waterlogging, a condition that leads to rhizosphere hypoxia and substantially impairs their growth and development. The response of peach to rhizosphere hypoxia is an intricate process regulated by the interplay of multiple genes. In our previous research, we investigated the optimal concentration of melatonin for peach seedlings subjected to waterlogging, revealing that a 200 μM treatment was most effective in enhancing survival for over 10 days under such stress conditions (Gu et al., 2021). The present study utilized transcriptomic and proteomic approaches to pinpoint key DEGs and metabolic pathways that are vital for the response to waterlogging, as well as the regulatory effects of melatonin priming. The capacity of melatonin to mitigate waterlogging stress and the underlying mechanisms warrant further investigation. The results of this study establish a fundamental framework and constitute a valuable genetic resource for subsequent inquiries into the regulatory mechanisms at play. This research furthers our understanding of the multifaceted role of phytomelatonin in the plant response to waterlogging, thereby facilitating the development of strategies to enhance the resilience of peach and other crops to this abiotic stress.

## 2 Materials and methods

### 2.1 Plant materials and treatment

Peach (*P. persica* L. Batsch) Maotao seedlings sourced from the Institute of Horticulture, Zhejiang Academy of Agricultural Sciences, Hangzhou, Zhejiang Province, China (120°11′55.21″E, 30°18′28.35″N), were cultivated in plastic pots filled with autoclaved substratum consisting of peat, vermiculite, and perlite at a ratio of 9:3:1 (v:v:v). The plants were grown in a phytotron under controlled conditions: a temperature of 25°C during the day and 20°C at night, with a 16-hour light/8-hour dark cycle. The plants were watered every three days with half-strength Hoagland’s nutrient mixture. Once the plants had developed approximately ten leaves, eighty uniformly sized plants were selected and divided into two groups. One group was treated with 0 μM melatonin (no priming), while the other was treated with 200 μM melatonin (melatonin priming) on the foliage and rhizosphere until the solution drained from the pots. Melatonin was administered once daily for five consecutive days. Following the priming treatment, both the no-priming and melatonin-priming groups were further divided into two subgroups: one for normal conditions (CK and MT) and the other for waterlogging treatment (WL and MT_WL). The waterlogging treatment involved maintaining a water level 2 cm above the soil surface for 24 hours. After treatment, the root samples were carefully collected, washed with distilled water, and used to determine related indices. An additional portion of each root sample was frozen in liquid nitrogen and stored at -80°C for subsequent transcriptome and proteome sequencing. Each treatment included three biological replicates.

### 2.2 Determination of root dry weight and membrane permeability

To determine the mass of the roots under waterlogging conditions, entire root of five seedlings were first dried at 105°C for 30 min and then at 80°C until a constant weight was achieved for dry weight measurement. Membrane permeability was assessed by measuring the relative electrolyte conductivity. Root samples (0.5 g) were rinsed with distilled water and immersed in a test tube with 10 mL of distilled water. The tubes were soaked for 12 h and then tested for conductivity (EC1) via a conductivity meter (Model DDSJ-308A). The tubes were placed in boiling water for 20 min, cooled to room temperature, and tested again for conductivity (EC2). The experiment was repeated three times. The relative electrolyte conductivity was calculated as EC1/EC2 × 100%.

### 2.3 Determination of leaf ROS levels

For the hydrogen peroxide (H_2_O_2_) content assay, 0.1 g fresh root samples were homogenized in 1 mL cold acetone. The H_2_O_2_ content was determined according to the manufacturer’s instructions for the H_2_O_2_ assay kit (Solarbio, Beijing, China), and its absorbance was measured at 415 nm. The data are presented as the amount of H_2_O_2_ per gram of fresh root (μmol/g FW). The O_2_^-^ production rate was measured via an O_2_^-^ assay kit (Solarbio, Beijing, China). Three independent extractions were subjected for each treatment.

### 2.4 Transcriptome analysis

Total RNA was extracted from peach roots via the ethanol precipitation method with the CTAB-PBIOZOL reagent. The quality and concentration of total RNA were evaluated via a NanoDrop system and an Agilent 2100 bioanalyzer (Thermo Fisher Scientific, Massachusetts, USA). mRNA was further purified via magnetic beads with dT, and a cDNA library was synthesized via reverse transcription. After the library was constructed, the BGIseq500 platform (BGI, Shenzhen, China) was used to sequence 100 bp paired-end reads. The raw data were filtered to remove sequence adapters and low-quality reads. The cleaned data were stored in FASTQ format for subsequent bioinformatics analysis.

HISAT2 (v2.0.4) was used to align the cleaned reads to the *Prunus persica* genome v2.1 (https://phytozome-next.jgi.doe.gov/info/Ppersica_v2_1). These cleaned reads were subsequently aligned to the database containing known and newly discovered coding transcripts constructed by BGI via Bowtie2 (v2.2.5), and then RSEM (v1.1.12) was used to calculate the gene expression level. Gene expression was analysed via fragments per kilobase of exon per million mapped reads (FPKM) values. The criteria for screening DEGs were a P value ≤ 0.05, a false discovery rate (FDR) < 0.001, and a fold change ≥ 2 or ≤ 0.5. DESeq2 (v1.4.5) software was used to conduct differential expression analysis, and the Q value was set to ≤ 0.05. In addition, to better understand the phenotypic changes, GO and KEGG (Kanehisa and Goto, 2000) enrichment analyses were performed on the annotated DEGs, and strict correction (Q value ≤ 0.05) was implemented for the terms and pathways with high significance.

### 2.5 Proteome analysis

For data-independent acquisition (DIA) analysis, total proteins were extracted from peach roots via the phenol extraction method. Bradford quantification and SDS−PAGE were used to check the quality of protein extraction. Enzymatic hydrolysis was carried out using trypsin at a specific ratio and for a certain period of time. High-pH RP separation technology is used to mix and inject samples for chromatographic analysis. Nano-LC−MS/MS is used for DDA and DIA, and well-defined parameters such as the ion source voltage, scanning range and resolution are adopted. MaxQuant and Spectronaut™ are used for bioinformatics analysis to handle tasks such as data identification, spectral library construction, deconvolution and quality control. The significance of differentially expressed proteins (DEPs) was evaluated according to the predefined comparison groups and the linear mixed effect model. Two filtering criteria (fold change ≥ 2 or ≤ 0.5 and P value < 0.05) were used to screen out DEPs. In addition, various types of functional annotation analyses, such as GO and KEGG pathway functional annotation analysis and time series analysis, were also conducted. On the basis of the quantification results, DEPs between the comparison groups were identified, and functional enrichment analysis, protein−protein interaction analysis and subcellular localization analysis of the DEPs were subsequently performed.

### 2.6 *In silico* analysis of *cis*-acting elements

The upstream regulatory regions of the DEGs were retrieved from the peach genome database (*Prunus persica v2.1*, https://phytozome-next.jgi.doe.gov/info/Ppersica_v2_1). The *cis*-acting elements in the promoter region (2 kb upstream of the translation initiation site) of the DEGs were identified via PlantCARE.

### 2.7 qRT−PCR analysis

The total RNA used was the same as that used for the transcriptome analysis. cDNA was synthesized via a FastKing One Step RT−qPCR Kit (Probe) (TIANGEN, Beijing, China) with 1 μg of RNA in each reaction. The gene sequences were collected from the *Prunus persica* genome in the JGI Phytozome database. Beacon designer 7.0 was used to find the qRT−PCR primers (**Supplementary Table S1**). qRT−PCR was performed according to previously described methods (Gu et al., 2021). Three technical replicates were performed for each gene. The 2^−ΔΔCT^ method was used to calculate the relative expression level of genes compared with that of *PpACTIN*.

### 2.8 Statistical analysis

The experimental data were analysed via SPSS software (version 17.0) and Excel 2016. The results are expressed as the means ± SDs. Means were compared between four groups via one-way ANOVA and Tukey’s test. Different letters on the histograms between different treatments indicate a significant difference at a *P* value < 0.05.

## 3 Results

### 3.1 Plant growth and development of peach under waterlogging and melatonin priming

**Figure 1:**
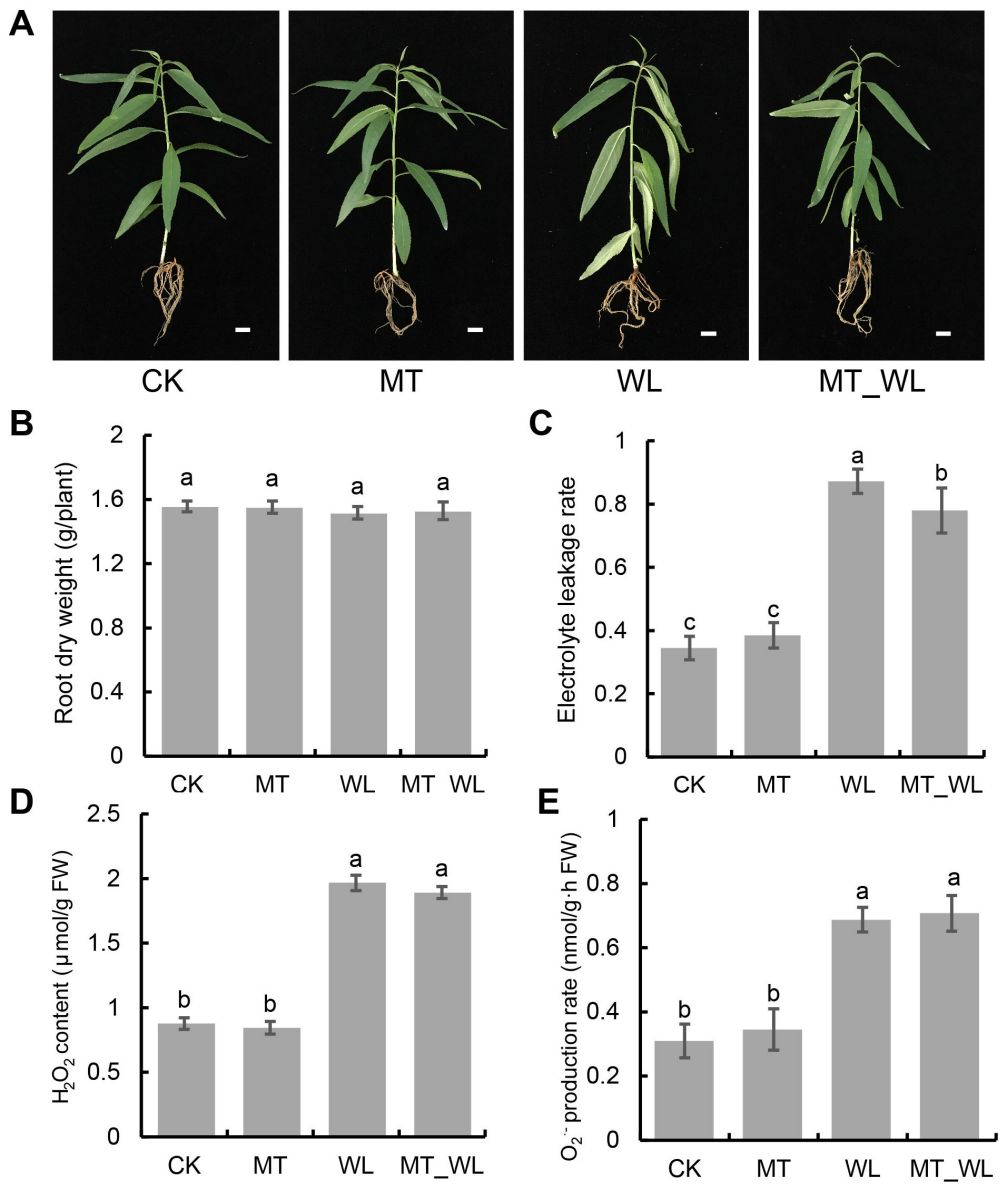
Effect of waterlogging and melatonin on morphological characteristics and physiological indexes of peach roots. **(A)** Morphological characteristics of entire plant. The scale bar was 1 cm. **(B)** Root dry weight. **(C)** Electrolyte leakage rate. **(D)** H_2_O_2_ accumulation. **(E)** O_2_^·-^ production rate. CK, control condition; MT, melatonin pretreated followed with control condition; WL, waterlogging treatment; MT_WL, melatonin pretreated followed with waterlogging treatment. The data are means ± SD of triplicate experiments. Different lowercase letters indicate that means are significantly different among the samples (*P* value < 0.05).

After a 24-h treatment duration, all the plantlets across the four treatment groups were meticulously rinsed and dried using clean tissue (**Figure 1A**). To evaluate the impact of waterlogging and melatonin priming on peach seedlings, various physiological parameters of the root system were examined after 24 h treatment, including root dry weight, electrical conductivity, H_2_O_2_ content and O_2_^·-^ production rate. The data indicated that dry root biomass was comparable across all four groups, with no statistically significant differences noted (**Figure 1B**). Nevertheless, the waterlogging treatment resulted in a substantial elevation in relative electrical conductivity, which was significantly mitigated by melatonin priming. This finding implies that melatonin exerts a protective effect on the cell membrane, diminishing the severity of damage (**Figure 1C**). Regarding ROS accumulation, the WL and MT_WL groups exhibited notably higher H_2_O_2_ contents and O_2_^·-^ production rates compared to the CK and MT groups. These results suggest that waterlogging triggers an increase in ROS levels, thereby intensify damage to the cell membranes of peach seedlings (**Figures 1D, E**).

### 3.2 Transcriptome sequencing and DEGs in response to waterlogging

**Figure 2:**
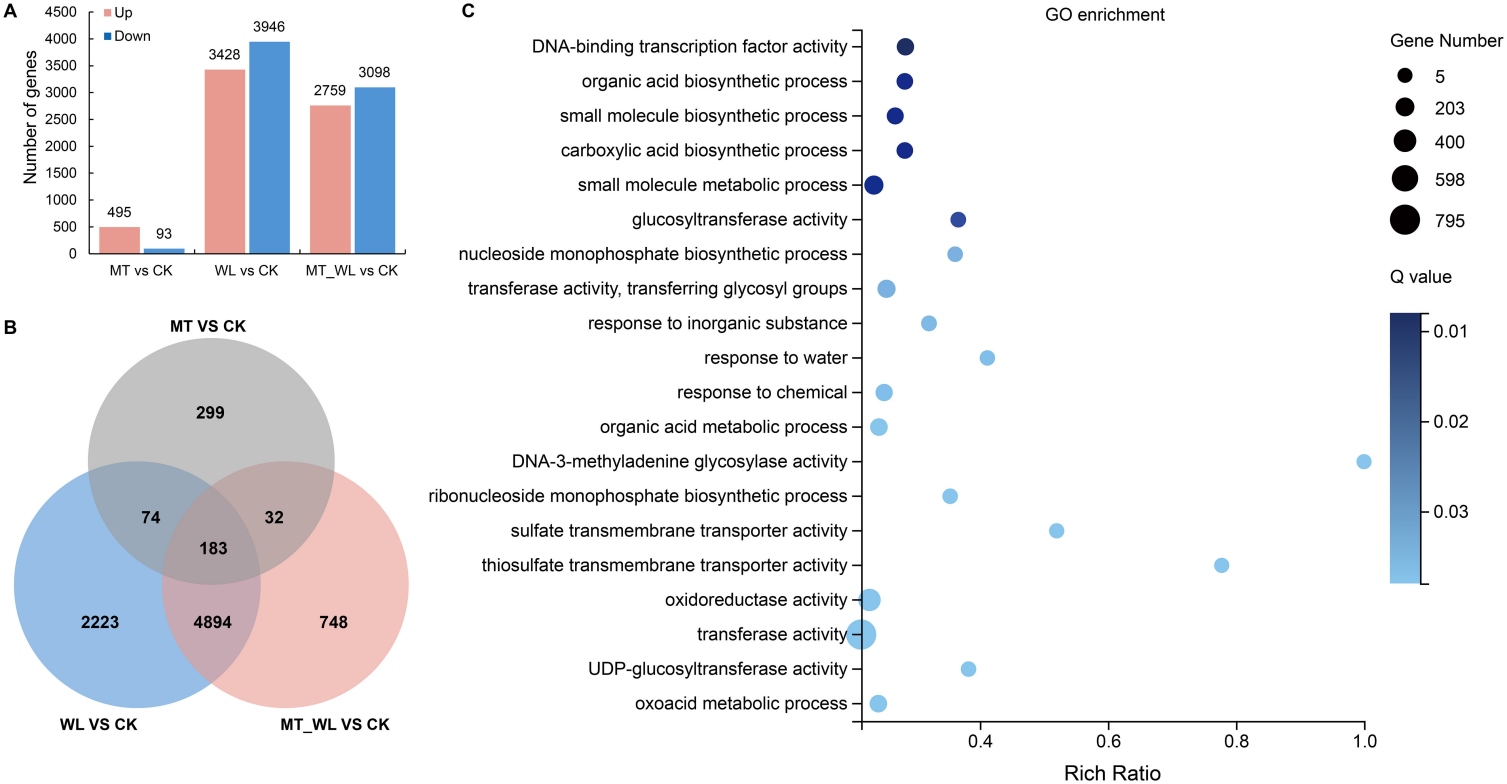
Analysis of DEGs. **(A)** The statistic of DEG number. The bar diagram reflected the number of up and down regulated DEGs in each comparison groups. **(B)** The Venn diagram of DEGs identified in the three comparison groups. **(C)** GO categories enrichment analysis of the chosen DEGs induced by both melatonin and waterlogging. The dot size indicates the number of DEGs, and different colors show different level. CK, control condition; MT, melatonin pretreated followed with control condition; WL, waterlogging treatment; MT_WL, melatonin pretreated followed with waterlogging treatment.

To investigate the mechanism of waterlogging response and melatonin priming protection on peach plants under rhizosphere flooding, we performed transcriptome sequencing analysis. RNA-seq (BioProject: PRJNA782223) was performed out on root samples from four treatment groups (CK, WL, MT, and MT_WL). We obtained more than 6.21 Gb of clean reads (Q20>96%; Q30>87%) from each sample, and subsequent to the refinement of low-quality reads and adapters, we achieved a mapping rate of over 87% to the *Prunus persica* reference genome. The preprocessing results, indicating the quality of the sequencing data, are presented in **Supplementary Table S2**. The results indicated that the RNA-seq data satisfied the quality criteria necessary for subsequent analysis. A total of 13819 DEGs with fold changes of ≥ 2 or ≤ 0.5 based on FDR < 0.05, were identified across all samples. In comparison to CK, 588 DEGs were detected in MT, with 495 genes upregulated and 93 genes downregulated. Relative to CK, 7374 DEGs were identified in WL, of which 3428 were upregulated and 3946 were downregulated. Compared with CK, 5857 DEGs were identified in MT_WL, including 2759 upregulated and 3098 downregulated genes (**Figure 2A**; **Supplementary Table S2**).

### 3.3 Analyses and expression verification of differentially expressed transcription factors

In **Figure 2B**, 183 DEGs were identified in peach roots under separate or combined treatments of melatonin and waterlogging. Additionally, 4894 DEGs were common to the WL vs CK and MT_WL vs CK comparisons. That is to say, a cumulative total of 5077 DEGs were induced by waterlogging, with or without the priming effect of melatonin. The top 20 most significantly enriched GO terms within the biological process category are depicted in **Figure 2C**. The genes were found to be significantly enriched for ‘DNA-binding transcription factor activity’ (129), ‘biosynthetic process’ (84), and ‘glucosyltransferase activity’ (37).

**Figure 3:**
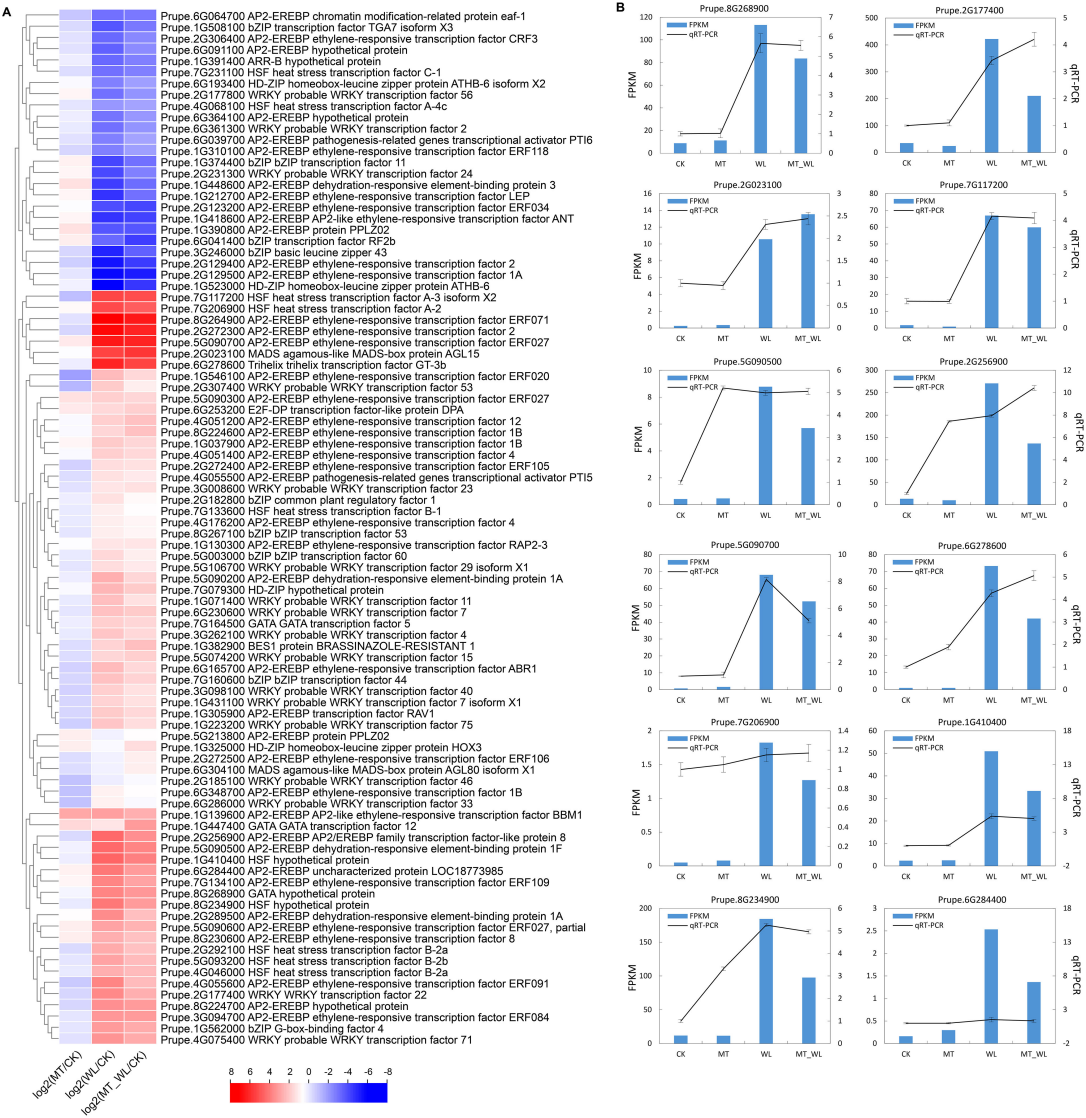
The expression levels of DEGs related to DNA-binding transcription factor activity. **(A)** Cluster heatmap of DEGs from RNA-seq. The color scale on the right represents the value of log2(Fold change). Red indicates upregulated and blue indicates down regulated. **(B)** Verification of DEGs related to DNA-binding transcription factor activity via qRT-PCR.

Within the ‘DNA-binding transcription factor activity’ category, 92 annotated transcription factors were found using a nonredundant database, encompassing 43 AP2/ERF, 17 WRKY, 10 HSF, 9 bZIP, 4 HD-ZIP, 3 GATA, and 2 MADS genes. The majority of these transcription factors are known to respond to abiotic stress. In both the WL and MT_WL groups, 67 genes were upregulated, while 25 were downregulated. Notably, a greater number of AP2/ERFs (30 upregulated, 13 downregulated) exhibited differential expression following waterlogging irrespective of melatonin pretreatment. The expression of the ethylene-responsive transcription factor Prupe.8G264900, was significantly elevated, reaching 7.1- and 6.5-fold increase relative to the control group, respectively, after waterlogging and melatonin pretreatment followed by waterlogging (**Figure 3A**). Among the downregulated genes, the homeobox-leucine zipper protein ATHB-6 was repressed in both the WL and MT_WL groups.

To assess the reliability of the RNA-seq data, a validation process was performed via qRT−PCR for 12 DEGs (**Figure 3B**). The FPKM values represent the relative transcript abundance of genes identified by RNA-seq. The FPKM values for the 12 DEGs suggest that these genes exhibit a high level of inducible expression in response to waterlogging. A comparison of the qRT−PCR and the FPKM data indicated that a significant number of DEGs displayed consistent expression profiles. The concordance between the qRT-PCR and RNA-seq reinforces the credibility of the RNA-seq data and its applicability for elucidating the genetic responses of peach seedlings to waterlogging.

### 3.4 DEGs involved in glycolysis/gluconeogenesis

**Figure 4:**
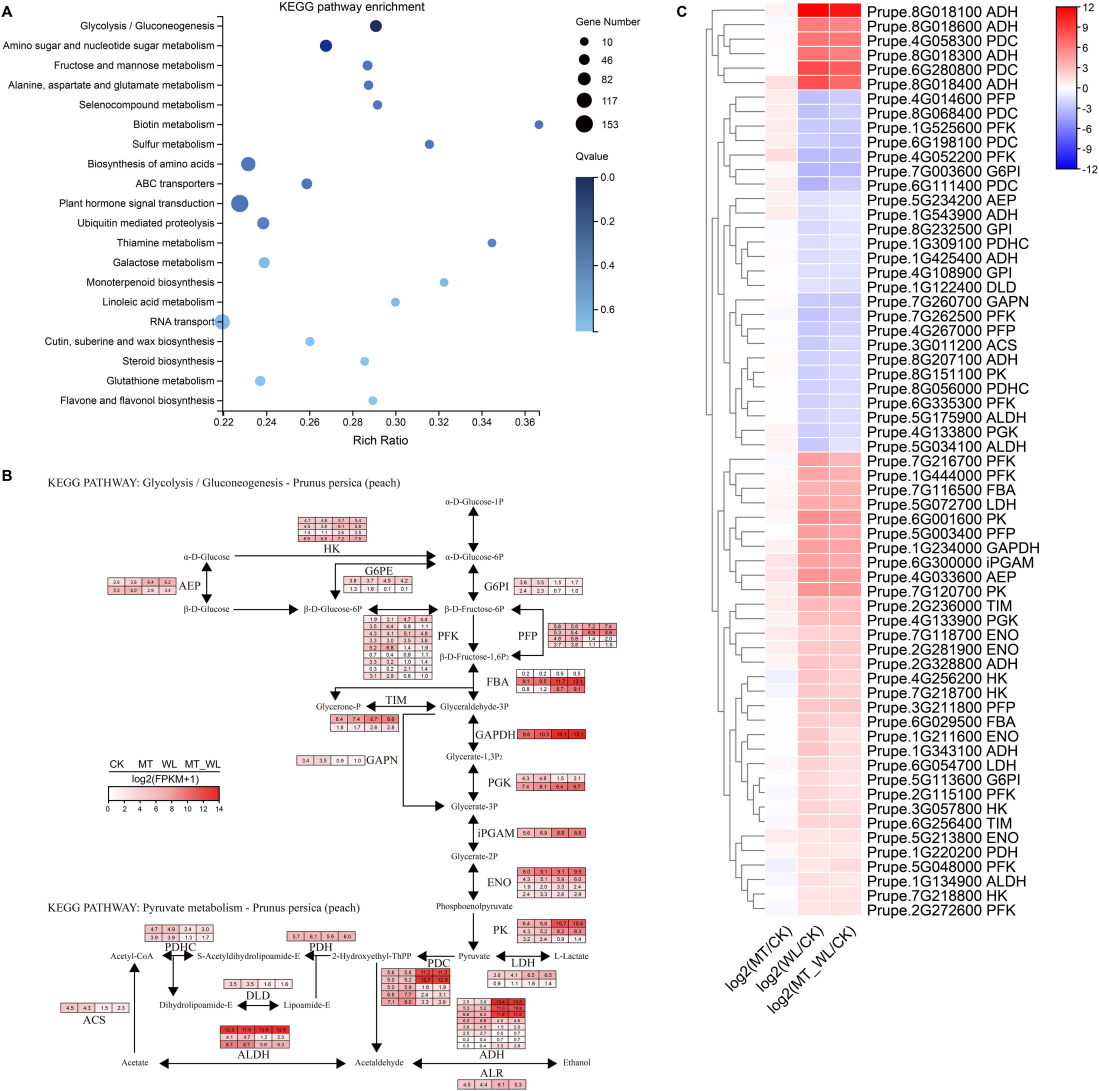
The key pathway induced by both melatonin and waterlogging. **(A)** KEGG pathway enrichment analysis of the chosen DEGs. The dot size indicates the number of DEGs, and different colors show different level. **(B)** The relative changes of key genes in Glycolysis/Gluconeogenesis pathway. The color scale represents the value of log2(FPKM+1) in four groups. **(C)** Cluster heatmap of DEGs from Glycolysis/Gluconeogenesis pathway. The color scale on the right represents the value of log2(Fold change). Red indicates upregulated and blue indicates down regulated.

To gain insights into the metabolic pathways implicated in the response of peach to waterlogging, particularly with melatonin pretreatment, 5077 co-expressed DEGs were subjected to KEGG enrichment analysis. The top 20 enriched pathways, as listed, encompassed ‘Glycolysis/Gluconeogenesis’, ‘Amino sugar and nucleotide sugar metabolism’, ‘Fructose and mannose metabolism’, ‘Alanine, aspartate and glutamate metabolism’, and ‘Plant hormone signal transduction’ (**Figure 4A**; **Supplementary Figure S1**). Furthermore, the glycolysis/gluconeogenesis (KEGG entry: map00010) was the most significantly enriched and comprised 64 DEGs, indicating its pivotal role in the metabolic response to waterlogging. As depicted in **Figure 4B**, the relative transcript levels of the differentially expressed enzymes within the glycolysis/gluconeogenesis pathway were normalized to Log2(FPKM+1). The 64 DEGs exhibited basal expression levels, and enzymes involved in downstream functions, including pyruvate kinase (EC 2.7.1.40, PK), pyruvate decarboxylase (EC 4.1.1.1, PDC), and alcohol dehydrogenase (EC 1.1.1.1, ADH), showed elevated expression levels. The metabolic processes catalyzed by ADH and PDC can produce energy, thereby providing essential support for survival under waterlogging.

Among the 64 DEGs associated with the glycolysis/gluconeogenesis pathway, 39 were upregulated in both WL and MT_WL relative to the CK, while 25 were downregulated (**Figure 4C**). The gene Prupe.8G018100 encoding ADH, exhibited the highest expression level, with Log2FC values of 12.0 and 11.0 in WL and MT_WL, respectively. Similarly, the expression levels of DEGs in the melatonin pretreatment group was lower compared to the control group. During the initial phase of waterlogging, glycolysis facilitates the production of a limited amount of energy to sustain plant metabolism and survival. Nevertheless, prolonged stress duration leads to the accumulation of glycolytic by-products, including ethanol and acetic acid, which can be detrimental to plant tissues. Melatonin pretreatment mitigates the exacerbation of damage by inhibiting excessive glycolytic reactions.

### 3.5 Proteomic analysis of DEPs in response to waterlogging

**Figure 5:**
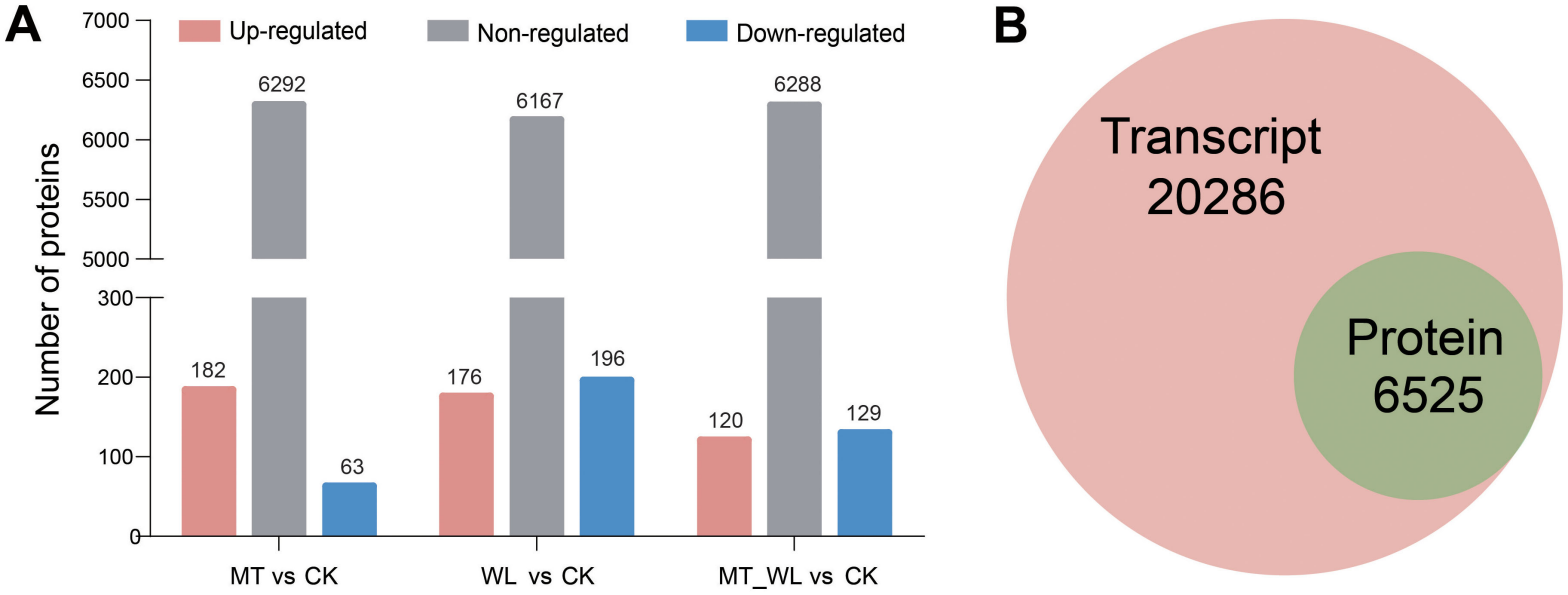
Analysis of DEPs. **(A)** Total number of DEPs identified in the three comparisons. Red bars represent proteins with upregulated, blue bars represent downregulated, and grey bars represent non-regulated. **(B)** Congruency of the detected transcriptome and proteome in different groups. CK, control condition; MT, melatonin pretreated followed with control condition; WL, waterlogging treatment; MT_WL, melatonin pretreated followed with waterlogging treatment.

To delineate the DEPs associated with melatonin and waterlogging, proteomics analyses were conducted on the same samples used for RNA-seq (CK, MT, WL and MT_WL). A total of 48720 unique peptides were identified, corresponding to 8614 unique proteins across the four samples. **Figure 5A** illustrates the variation in protein expression following melatonin and waterlogging treatments. A total of 245 specifically expressed proteins were significantly different between MT and CK groups, with 182 upregulated and 63 downregulated. In the comparison of WL and CK groups, 176 proteins were upregulated, and 196 were upregulated. The comparison of MT_WL vs CK revealed 120 upregulated proteins and 129 downregulated proteins (**Supplementary Table S3**). The expression of the majority of proteins, exceeding 6000 in number, did not exhibit significant differences. Under waterlogging, the number of downregulated proteins surpassed that of upregulated proteins in the samples. All coding transcripts corresponding to the 6525 identified proteins were present within the 26811 transcripts identified by RNA-seq, indicating that transcripts were detected for each identified protein (**Figure 5B**). For functional annotation, the identified proteins were cross-referenced with the GO and KEGG databases. The GO database was utilized to predict potential functions and classify proteins functionally. The results revealed that the ‘catalytic activity’ of MFs, the ‘cell’ of CCs and the ‘metabolic process’ of BPs were the most significantly enriched in response to melatonin and waterlogging treatments (**Supplementary Figure S2**, **Supplementary Table S4**). Proteins within the ‘carbohydrate transport and metabolism’ category were predominantly involved in maintaining active metabolic processes. Proteins typically operate in conjunction with one another, engaging in various biological functions. KEGG pathway enrichment analysis was employed to construct a protein interaction network (**Supplementary Figure S3**, **Supplementary Table S5**). The DEPs were predominantly enriched within the metabolic categories ‘Global and overview maps’ and ‘Carbohydrate metabolism’, aligning with the RNA-seq findings.

### 3.6 Conjoint analysis of DEGs and DEPs

**Figure 6:**
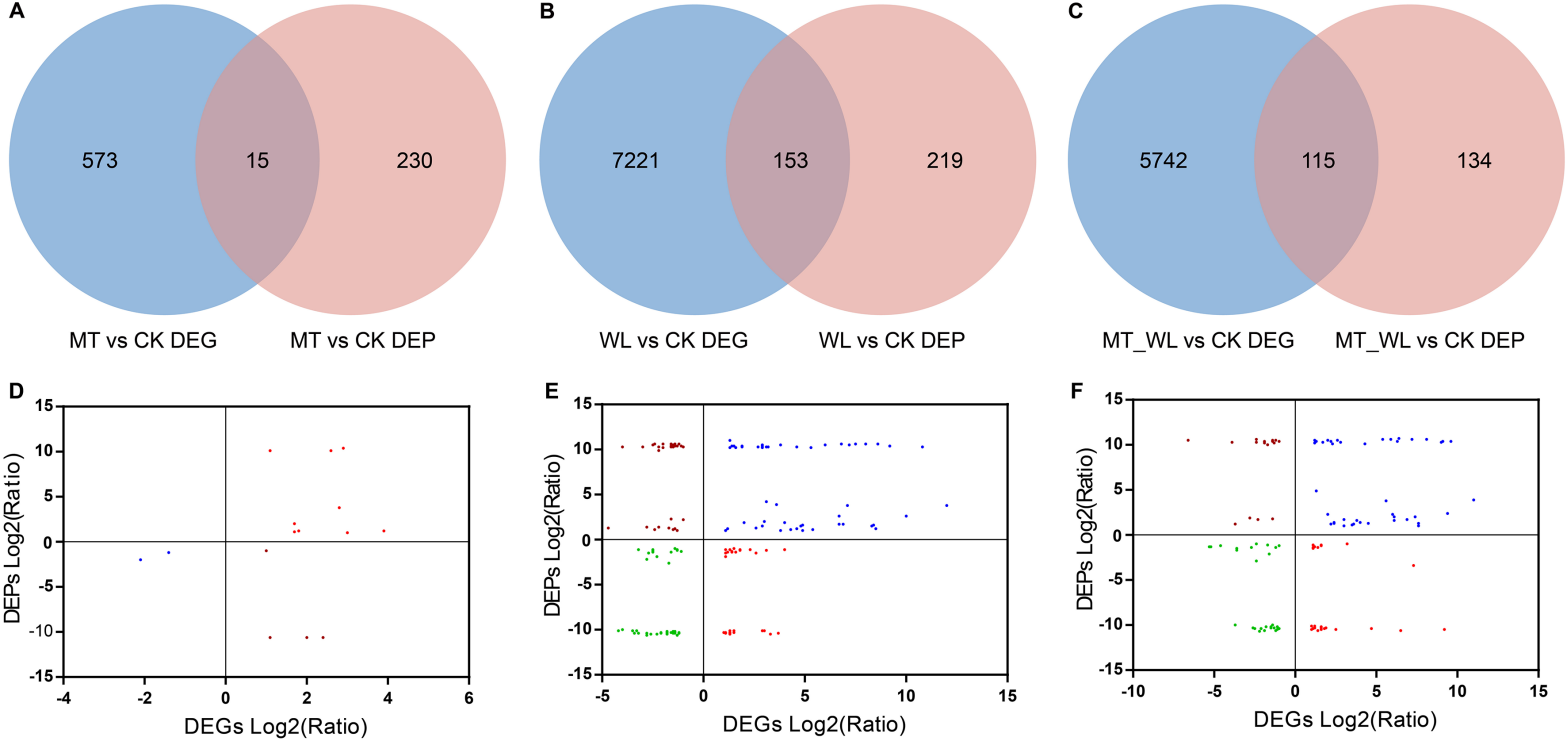
Conjoint analysis of DEGs and DEPs in response to waterlogging and melatonin. The Venn diagram of DEGs and DEPs identified in comparison MT vs CK **(A)**, WL vs CK **(B)**, and MT_WL vs CK **(C)**. Comparison of changes in protein and cognate mRNA abundance levels in comparison MT vs CK **(D)**, WL vs CK **(E)**, and MT_WL vs CK **(F)**. CK, control condition; MT, melatonin pretreated followed with control condition; WL, waterlogging treatment; MT_WL, melatonin pretreated followed with waterlogging treatment.

A combined analysis of DEGs and DEPs during waterlogging was conducted. To investigate the concordance between the differential expression levels of transcripts and proteins, Venn diagrams revealed 818 co-expressed genes and proteins in response to melatonin pretreatment, which 15 being both DEGs and DEPs (**Figure 6A**). Following waterlogging, 7593 genes were co-expressed with corresponding proteins, which 153 being both DEGs and DEPs (**Figure 6B**). When waterlogging was performed following melatonin pretreatment, there were 5991 co-expressed genes and proteins, of which 115 were both DEGs and DEPs (**Figure 6C**).

**Figure 7:**
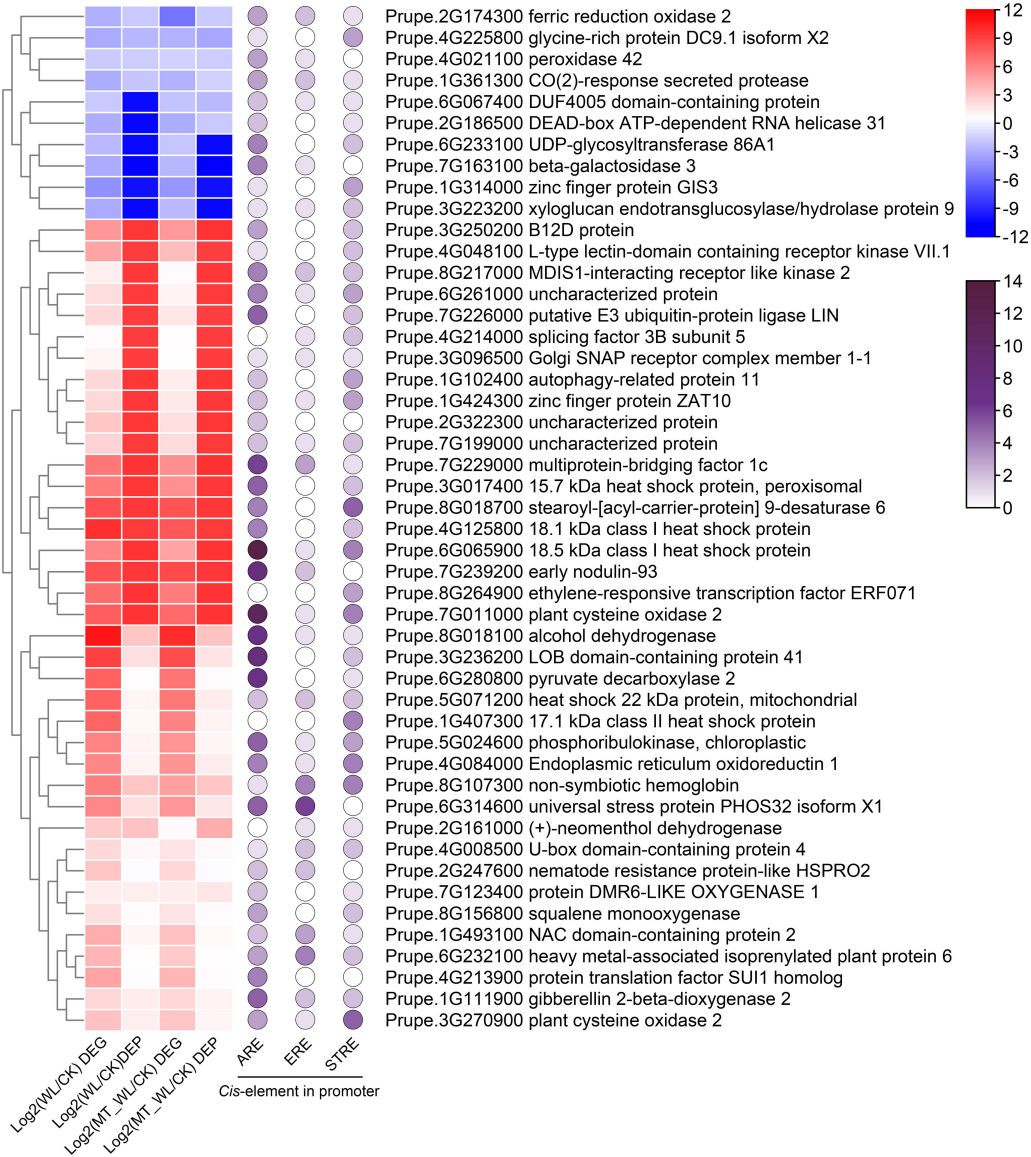
Core DEGs/DEPs in conjoint analysis of transcriptome and proteome. The expression heatmap and *cis*-element in the promoter region of common DEGs and DEPs in different comparison groups. The color scale on the right represents the value of log2(Fold change). Red indicates upregulated and blue indicates down regulated. Purple cycles indicate the number of waterlogging related *cis*-elements.

Additionally, we performed a correlation analysis between the common DEGs and DEPs to determine the co-expression relationships under each condition. Initially, among the 15 common DEGs and DEPs in the MT vs CK comparison, 11 were positively correlated (**Figure 6D**). For the WL vs CK and MT_WL vs CK, 61.4% (94/153) and 61.7% (71/115) of the common genes/proteins presented the same expression tendencies, respectively (**Figures 6E, F**). The coincidence of expression trends among many common genes/proteins suggests that a significant number of genes are regulated at both the transcriptional and translational levels. Under waterlogging stress, a higher number of DEGs and DEPs exhibited greater fold changes compared to normal conditions. Moreover, of the 94 and 71 DEGs/DEPs with consistent expression trends in WL vs CK and MT_WL vs CK, 48 were common to all samples, indicating that these genes and proteins play special roles in response to waterlogging and melatonin media tolerance (**Figure 7**).

A combined analysis of the transcriptome and proteome identified 48 core DEGs/DEPs. 5 heat shock proteins were upregulated across all the groups (**Figure 7**). Additionally, among the stress-related proteins, those involved in hypoxia response, including the ethylene-responsive transcription factor ERF071, plant cysteine oxidase 2, alcohol dehydrogenase, and pyruvate decarboxylase 2, were found in the core members. Analysis of *cis*-elements in the promoter regions showed that one or more of the three hypoxia-related *cis*-elements, ‘anaerobic induction’, ‘ethylene response’, and ‘stress response’, were present in the promoters of the 48 genes. Of the 48 DEGs, 91.7% contained anaerobic induction elements, indicating that majority of these DEGs/DEPs are inducible by the low-oxygen conditions associated with waterlogging. The range of hypoxia-related *cis*-elements in these genes varies from 2 to 18. Notably, Prupe.6G065900, which encodes a 18.5 kDa class I heat shock protein, possesses 13 anaerobic induction elements, 1 ethylene response element, and 4 stress response elements. Similarly, Prupe.7G011000, which encodes plant cysteine oxidase 2, contains 16 *cis*-elements.

## 4 Discussion

Abiotic stresses, such as extreme temperatures (cold and heat), water imbalances (drought and waterlogging), salinity, and heavy metal exposure, markedly impede plant growth and development. The peach, celebrated for its exquisite taste, nutritional benefits, and broad appeal, is extensively cultivated in China (Li and Wang, 2020). Nevertheless, peach trees are especially susceptible to waterlogging due to their shallow root systems, which are highly oxygen-dependent and profoundly impacted by the hypoxic conditions of waterlogged soils. Waterlogging represents a significant impediment to the peach industry in southern China (Tian et al., 2021). Elucidating the regulatory mechanisms behind peach seedling responses to waterlogging is vital for comprehending plant adaptation in rainy environments. This insight can be leveraged to develop waterlogging-tolerant *Prunus* germplasms, thereby improving peach yield under challenging conditions (Ateeq et al., 2023). In this research, we utilized RNA-seq and DIA proteomic analyses to pinpoint genes and proteins involved in the response to melatonin and waterlogging. By integrating multiomics data, we conducted a systematic examination of peach roots to establish correlations between mRNA and protein expression. As a result, we identified DEGs and DEPs associated with waterlogging responses in peach.

The number of DEGs in the MT vs CK was lower compared to the other two comparisons, with the WL vs CK exhibiting the highest number of DEGs, in agreement with the proteomic data. These results imply that melatonin pretreatment induced a relatively modest differential gene expression, whereas waterlogging, representing a more severe stressor, triggered a substantially larger number of DEGs. This suggests that melatonin pretreatment may have mitigated the necessity for a robust activation of stress-responsive genes in response to subsequent waterlogging stress. Recent transcriptomic studies have illuminated the involvement of various metabolic pathways, including ‘global and overview maps’, ‘carbohydrate metabolism’, ‘amino acid metabolism’, and ‘energy metabolism’, in the responses to waterlogging (Kuai et al., 2016; Zhang et al., 2022) as well as in response to other abiotic stresses (Zhou D. et al., 2020). In the current study, KEGG annotation identified that the DEGs were mainly grouped in pathways associated with ‘global and overview maps’, ‘carbohydrate metabolism’, and ‘environmental adaptation’. These observations imply that waterlogging may affect the metabolism of soluble sugars, organic acids, and phenols in peach.

Based on Venn diagram analysis, 92 annotated transcription factors were identified, including members of the AP2/ERF family, which are prominent activated by flooding in plants (Mustroph et al., 2009; Wang et al., 2021). Previous research has demonstrated that AP2/ERF transcription factors play critical roles in regulating fermentation and ROS signalling by modulating the expression of genes such as *ADH*, *PDC*, *RBOHD*, *HRU1* (Wang K. et al., 2024). Additionally, members of the ERF subfamily within group VII, which are involved in hypoxia response, have been functionally analysed in Arabidopsis and rice (Gasch et al., 2016; Lin C. et al., 2019; Weits et al., 2021). In the present study, the three most highly expressed transcription factors, Prupe.8G264900, Prupe.2G272300, and Prupe.5G090700, were classified as ERF transcription factors (**Figure 3A**). Furthermore, Prupe.8G264900 (ERF071) was identified as an ERF VII member (**Supplementary Table S6**) (Zhao et al., 2018), highlighting the significance of ERF VII members in regulating the response of peach to waterlogging. In Arabidopsis, ERF VIIs have been identified as regulators of hypoxia survival responses, mediating the increase activities of PDC and ADH, which facilitate the transition from aerobic to anaerobic metabolism (Gibbs et al., 2011). Studies indicate that the upregulation of specific ERFVII transcription factors can induce the activation of genes such as *LOB41*, *ADH1/2*, and *PDC2*, involved in alcoholic fermentation and scavenge ROS, thereby enhancing waterlogging tolerance in kiwifruit (Pan et al., 2019; Liu et al., 2022; Bai et al., 2023). Consequently, in response to waterlogging signals like ethylene and hypoxia, ERF VIIs rapidly accumulate and initiate a signalling pathway that promotes tolerance to waterlogging.

In addition to AP2/ERF family, WRKY transcription factors are crucial for integrating environmental cues and influencing plant growth under stress conditions. In our study, 14 out of the 17 WRKY genes were upregulated in response to waterlogging, indicating a significant role for WRKYs in the waterlogging response of peach. Previous research on *Phoebe bournei* has shown that WRKY members are highly responsive to waterlogging, with *PbWRKY36* identified as a candidate gene potentially plays a pivotal role in enhancing resilience to waterlogging (Wang Z. et al., 2024). Other studies have demonstrated that WRKYs act as positive regulators of hypoxia responses, such as at the onset of waterlogging in sesame (Wang et al., 2021) and submergence in rice (Viana et al., 2018). Moreover, the heterologous expression of the sunflower transcription factor *HaWRKY76* in Arabidopsis was shown to confer submergence tolerance with preserved carbohydrate and increased cell membrane stability (Raineri et al., 2015).

Together, the robust expression of ERFs and WRKYs revealed in our study that these transcription factors are involved in the response to waterlogging in peach. Previous research by Gao indicated that the WRKY22/33 and AP2-EREBP genes may be implicated in antioxidant defense and enhance waterlogging tolerance in kiwifruit (Gao et al., 2022). The transcriptional alterations induced by waterlogging are predominantly mediated by the ERF and WRKY transcription factors. Nonetheless, it is yet to be determined whether these two gene families interact within the same signalling pathways during waterlogging. Exploring the potential interplay between WRKYs and ERFs in the context of waterlogging responses is a subject that deserves additional experimental investigation.

The KEGG pathway enrichment analysis of coregulated genes indicated that DEGs under waterlogging stress and melatonin priming were significantly enriched in pathways associated to ‘glycolysis/gluconeogenesis’, ‘Starch and sucrose metabolism’, and ‘plant hormone signal transduction’ (**Figure 4A**; **Supplementary Figure S1**). Beyond several commonly regulated expression pathways, glycolysis/gluconeogenesis exhibited the most pronounced response to waterlogging. Typically, plants depend on glycolysis and ethanol fermentation as alternative energy sources during the energy deficit induced by waterlogging (Zhou W. et al., 2020; Habibi et al., 2023). The expression of most genes encoding key enzymes in the glycolysis/gluconeogenesis pathway was upregulated compared to the control, with marked increases in fold change. These expression patterns are align with previous findings that underscore the importance of glycolysis/gluconeogenesis in the transcriptional regulation and metabolic adaptation of sweet cherry (Michailidis et al., 2021) and Chinese bayberry (Jiao et al., 2023). Furthermore, the enhanced expression of specific energy metabolism pathways aids peach in better managing waterlogging (Ateeq et al., 2023). This adaptive strategy reinforces the plant’s ability to withstand waterlogging, thereby enhancing survival.

Due to ATP depletion under waterlogging stress, soluble sugars are transported to the roots to furnish substrates for glycolysis and anaerobic respiration. Glycolysis maintains a sufficient supply of carbohydrates, enabling plants to endure hypoxic conditions (Habibi et al., 2023). In our study, both gene transcripts and metabolites, including ADH, PDC, PK, 6-phosphofructokinase (PFK), diphosphate-dependent phosphofructokinase (PFP), and glyceraldehyde 3-phosphate dehydrogenase (GAPDH), were significantly enriched in the glycolysis/gluconeogenesis and pyruvate metabolism pathways during waterlogging. These results suggest that the response of peach to waterlogging is closely associated with respiratory metabolism. Anaerobic metabolism is sustained through the activation of fermentative pathways, which support ATP production via the glycolytic route. Glycolysis can proceed with NADH oxidation through lactic acid and ethanol fermentation from pyruvate (Bailey-Serres and Voesenek, 2008). Nevertheless, the fermentation pathway can be detrimental to plant cells due to the production of lactic acid and acetaldehyde (van Dongen and Licausi, 2015). The onset of ethanol fermentation involves inhibition of lactate dehydrogenase (LDH) activity and the stimulation of PDC. Transcripts of four ADH genes (Prupe.8G018100, Prupe.8G018300, Prupe.8G018400, and Prupe.8G018600) and two PDC genes (Prupe.4G058300 and Prupe.6G280800) were upregulated by waterlogging. However, melatonin priming in peach led to reduced expression levels of ADH, PDC, and other fermentation-related genes, indicating a regulatory role for melatonin in modulating the anaerobic metabolism pathway before waterlogging.

Although the multifunctional roles of melatonin in plants have been extensively studied, comprehensive transcriptomic and proteomic profiling of Prunus genotypes under stress conditions remains scarce. In our previous work, we demonstrated that 200 μM melatonin effectively enhanced the tolerance of peach plantlets to waterlogging by reducing lipid membrane peroxidation while also enhancing the antioxidant system (Gu et al., 2021). Building on these findings, subsequent omics analyses can elucidate the underlying regulatory mechanisms. Transcriptomic sequencing of *Clematis* varieties under waterlogging was performed to examine the effects of melatonin on waterlogging tolerance, revealing that melatonin enhanced flooding tolerance in *Clematis* by improving photosynthetic efficiency and antioxidant enzyme activity (Chen et al., 2024). In tomato, appropriate melatonin application significantly enhanced tolerance to low night temperatures, as evidenced by transcriptomic and proteomic approaches (Yang et al., 2022). In drought-stressed wheat treated with 100 μM melatonin, integrative physiological, transcriptomic, and proteomic data analyses indicated that melatonin modulated JA related genes, transcription factors, and starch and sucrose metabolism genes, thereby enhancing wheat drought tolerance (Luo et al., 2023). In apple, melatonin positively contributes to the accumulation of plant dry weight by upregulating genes associated with N cycling and significantly augmented the abundance of beneficial bacteria in the rhizosphere, facilitating the recovery of apple following waterlogging (Cao et al., 2024a). In the current study, through the combination of transcriptomic and proteomic analyses, we identified that more stress-responsive genes, such as ERF071, PCO2, HSP, and ATG11, were upregulated by waterlogging at both the transcriptional and protein levels. However, under melatonin priming, the expression levels of these genes did not exceed those observed under waterlogging alone, and protein levels remained comparable. Melatonin pretreatment may alter the internal physiological equilibrium of plants, prompting the adoption of alternative coping strategies to waterlogging. Peach pretreated with melatonin may prioritize energy allocation for maintaining the stability of fundamental cell metabolism over the induction of numerous stress-related genes. This could involve protective mechanisms that prevent excessive activation of these genes or proteins.

Significant challenges relate to the scalability of melatonin priming in field applications. In a controlled laboratory milieu, the precise delivery of melatonin and the monitoring of its effects are relatively straightforward. However, scaling up to field-level operations introduces a layer of complexity. Variations in soil types, fluctuating weather patterns, and differences in plant populations can all influence the effectiveness of melatonin priming. Moreover, the financial implications of applying melatonin across extensive agricultural areas could be daunting, which might limit its broader implementation. Consequently, additional research is essential to address both the practical application of melatonin in field settings and the genetic improvement of melatonin-associated genes to enhance plants’ ability to cope with stress.

## 5 Conclusion

**Figure 8:**
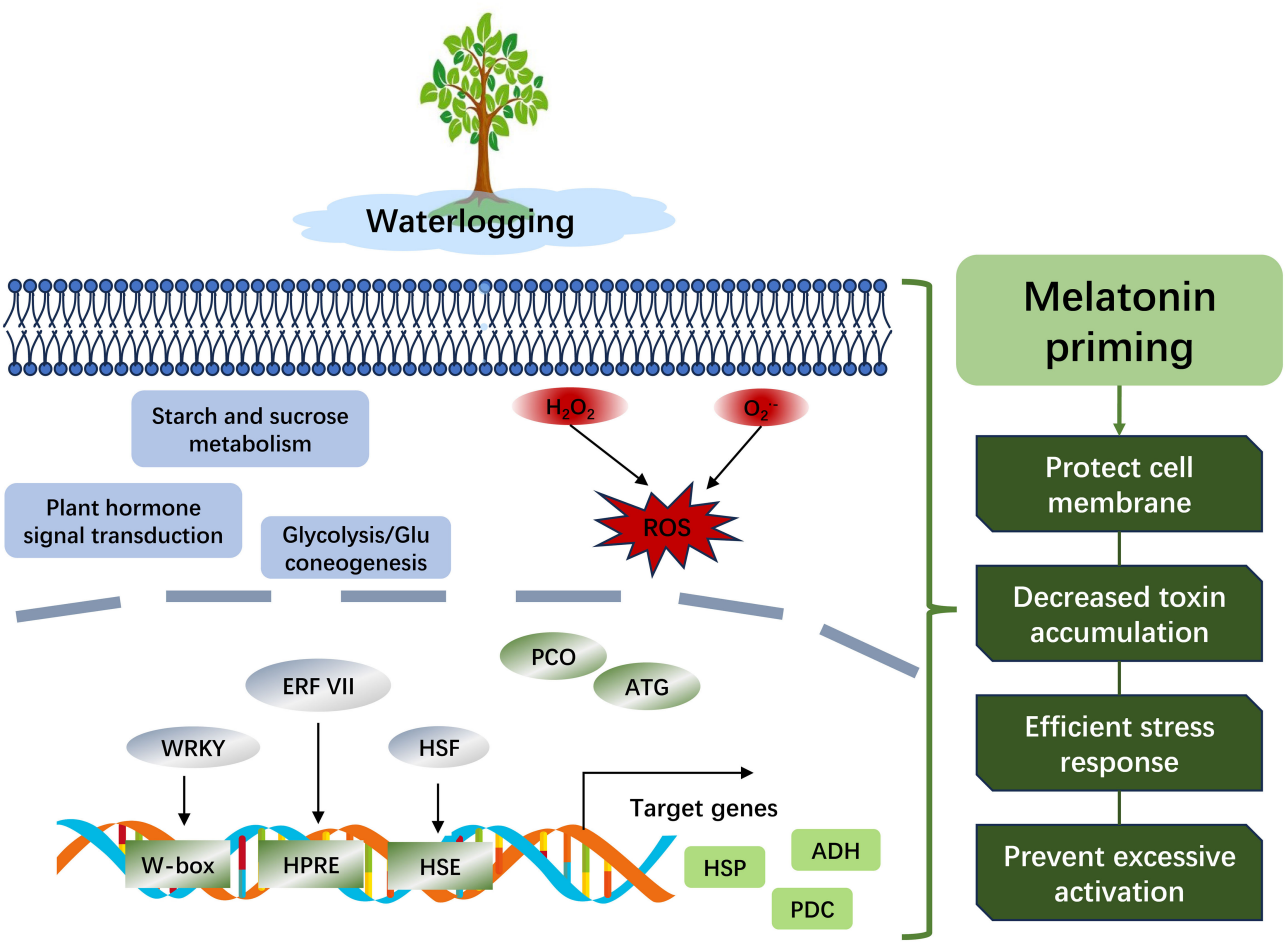
A hypothetical model of waterlogging response and melatonin priming mechanisms in peach.

In summary, waterlogging stress adversely affects the membrane system and oxidative balance in peach, while melatonin facilitates growth recovery and modulates the waterlogging response regulation (**Figure 8**). Transcriptomic and proteomic analyses indicated that the number of upregulated DEGs was fewer compared to downregulated ones following waterlogging, and exogenous melatonin prevented excessive activation of genes or proteins. Furthermore, transcription factors from the AP2/ERF and WRKY families were implicated in the response to waterlogging. The glycolysis/gluconeogenesis pathway has been identified as a pivotal pathway for peach to withstand waterlogging. The ERF VII family member ERF071 (Prupe.8G264900), ADH (Prupe.8G018100), and PCO (Prupe.7G011000) are promising candidates for manipulating the waterlogging response. Future research should aim to develop transgenic lines and perform biochemical assays to delineate their functions more precisely. Our findings provide actionable targets for breeding waterlogging-tolerant peach varieties and strategies to alleviate abiotic stress in horticulture.

## Data Availability

Data of transcriptome are available in NCBI, BioProject: PRJNA782223.

## Supplementary material

Supplementary Figure S1 KEGG pathway enrichment maps of DEGs and DEPs.

Supplementary Figure S2 GO enrichment of DEPs in the three comparisons.

Supplementary Figure S3 KEGG enrichment of DEPs in the three comparisons.

Supplementary Table S1 Primers for qRT-PCR.

Supplementary Table S2 Quality metrics of Transcriptome.

Supplementary Table S3 List of DEPs in comparison groups.

Supplementary Table S4 Differential GO enrichment of DEPs.

Supplementary Table S5 Differential KEGG enrichment of DEPs.

Supplementary Table S6 The three ERF-VII genes in peach and their characteristics.
